# Influence of the Near Molecular Vicinity on the Temperature Regulated Fluorescence Response of Poly(*N*-vinylcaprolactam)

**DOI:** 10.3390/polym8040109

**Published:** 2016-03-25

**Authors:** Anne Enzenberg, André Laschewsky, Christine Boeffel, Erik Wischerhoff

**Affiliations:** 1 Institute of Chemistry, University of Potsdam, Karl-Liebknecht-Str. 24-25, Potsdam-Golm D-14476, Germany; anne.enzenberg@gmail.de; 2 Fraunhofer Institute of Applied Polymer Research IAP, Geiselberg-Str. 69, Potsdam-Golm D-14476, Germany; christine.boeffel@iap.fraunhofer.de

**Keywords:** thermo-responsive polymers, poly(*N*-vinylcaprolactam), lower critical solution temperature, fluorescent dye monomers, naphthalimide, solvatochromism, polymeric sensors, molecular thermometers

## Abstract

A series of new fluorescent dye bearing monomers, including glycomonomers, based on maleamide and maleic esteramide was synthesized. The dye monomers were incorporated by radical copolymerization into thermo-responsive poly(*N*‑vinyl-caprolactam) that displays a lower critical solution temperature (LCST) in aqueous solution. The effects of the local molecular environment on the polymers’ luminescence, in particular on the fluorescence intensity and the extent of solvatochromism, were investigated below as well as above the phase transition. By attaching substituents of varying size and polarity in the close vicinity of the fluorophore, and by varying the spacer groups connecting the dyes to the polymer backbone, we explored the underlying structure–property relationships, in order to establish rules for successful sensor designs, e.g., for molecular thermometers. Most importantly, spacer groups of sufficient length separating the fluorophore from the polymer backbone proved to be crucial for obtaining pronounced temperature regulated fluorescence responses.

## 1. Introduction

Many nonionic polymers exhibit thermo-responsive behavior in aqueous solution, *i.e.*, they undergo a coil-to-globule collapse phase transition exhibiting a lower critical solution temperature (LCST) [1,2,3,4,5,6]. This phase transition is associated with the transformation of the polymer chains from a well hydrated, and thus expanded state to a shrunken collapsed one. Still in many cases, macroscopic phase separation is not observed, but phase separation is limited to the mesoscopic level due to the formation of stable colloidal aggregates in the 100–1000 nm range, the so-called “mesoglobules” [7].

Occasionally, such thermo-responsive polymers have been functionalized by incorporating fluorescence dyes. Beyond acting as tracers [8,9,10,11], the incorporated dyes have been used, on the one hand, to follow the phase transition more easily, or to elucidate the transition mechanism [12,13,14,15,16,17,18,19,20,21,22,23,24]. On the other hand, the combination of thermo-responsiveness and color in the same polymers has been explored for various sensor applications, e.g., for molecular thermometers [25,26,27,28,29,30,31,32,33,34,35,36,37,38,39,40,41,42,43,44] or for the detection of ions or biologically relevant molecules [45,46,47,48,49,50]. When passing through the phase transition, the environment of the polymer anchored dyes is changed so that the spectroscopic behavior may be altered in manifold ways. For instance, the extinction coefficient or the quantum yield may change, solvatochromism or changes of the overall shape of the spectra may occur, or the Förster resonance energy transfer (FRET) of incorporated donor-acceptor pairs may be switched on and off [44]. Such a thermo-responsive modulation of the system’s color differs inherently from the classical thermochromism of polymer systems that is based either on temperature induced changes of the chromophore structure, or on changes of the underlying supramolecular periodic structure in the case of structural color [51].

Recently, we have shown that 4-(*N*,*N*-dialkylamino)-1,8-naphthalimide fluorophores are well suited for indicating the passage through LCST-type phase transitions, when incorporated in water-soluble thermo-responsive polymers [21]. These fluorophores exhibit marked solvatochromism in their absorbance and emission spectra, as well as a modulation of the fluorescence quantum yield and intensity. However, while the effects were pronounced in the case of poly(*N*-isopropyl acrylamide) (PNIPAM), they were moderate only in the case of poly(diethyleneglycol-monomethylether acrylate) (PDEGA), and were marginal in the case of a copolymer of diethyleneglycol-monomethylether methacrylate and (oligoethyleneglycol)-monomethylether methacrylate (P(DEGMA-*co*-OEGMA)). Interestingly, a similar phenomenon was reported for the incorporation of the solvatochromic azo dye Disperse Red 1 into poly(diethyleneglycol-monomethylether methacrylate) (PDEGMA) and into copolymers of the P(DEGMA-*co*-OEGMA) family [34]. Whereas heating through the phase transition resulted in a pronounced solvatochromism of the absorbance spectra for PDEGMA, which thus may be used as molecular thermometer, no color shift could be observed for the copolymer.

For the use of such dye-functionalized thermo-responsive polymers, e.g., for designing effective and sensitive sensor systems based on the phase transition induced spectroscopic changes, the understanding of this phenomenon is crucial. When considering possible reasons for the striking differences between the spectroscopic behavior of the various polymers, several explanations have been invoked [21]. These include the different extents of dehydration above the phase transition of the various polymers, as well as their ability or inability, respectively, for inter- and intramolecular hydrogen bonding. The latter should favor densification of the collapsed polymer chains in the case of PNIPAM, for instance. Still, the various explanations have remained speculative. Alternative molecular factors contributing to the temperature-dependent spectral responses might be the different polarities or interactions of the amide and ether moieties that provide the polymers’ hydrophilicity, or differing features of the acrylic and methacrylic polymer backbones, such as reduced mobility or increased hydrophobicity of the latter.

**Figure 1:**
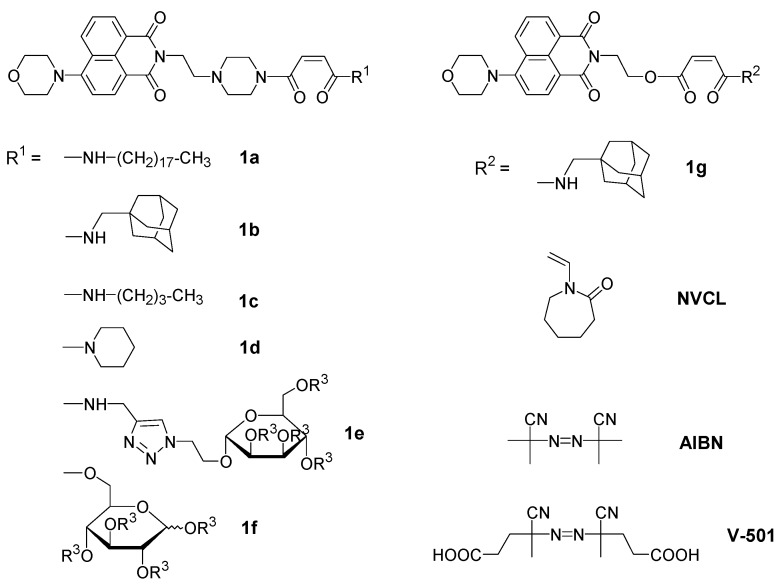
Chemical formula of the dye monomers synthesized, and the monomers and initiators used in the synthesis of fluorophore-functionalized thermo-sensitive polymers. For monomers **1e** and **1f**, R^3^ = acetyl, while, for polymers **PNVCL-e** and **PNVCL-f**, R^3^ = H.

We have therefore pursued the question, how to design appropriate fluorescent polymer molecular thermometers for aqueous systems, by creating a series of thermo-sensitive polymers functionalized by a solvatochromic fluorophore of the 4-(*N*,*N*-dialkylamino)-1,8-naphthalimide type (Figure 1). The polymers are based on poly(*N*-vinylcaprolactam) (PNVCL), which has been shown to exhibit a LCST in aqueous solution in the physiologically interesting range of 30–40 °C similar to the aforementioned thermo-responsive polymers, exhibiting typical Flory–Huggins demixing (“Type I behavior”) [52,53,54,55,56]. Characteristically for that, both the critical concentration as well as the LCST decrease markedly with increasing molar mass [52,54,57,58,59], in contrast to the behavior of PNIPAM (“Type II behavior”) [3]. Accordingly, PNVCL combines features of PNIPAM, such as the amide-promoted hydrophilicity, with features of the PDEGA/PDEGMA/POEGMA families, such as LCST behavior of type I. In addition, as the latter polymers, PNVCL is considered to be more biocompatible than PNIPAM [60]. Concerning the nature of the polymer backbone, PNVCL belongs to the vinyl polymer family, as PNIPAM and PDEGA do, while PDEGMA and POEGMA represent sterically much more crowded vinylidene polymers. However, different from all the other thermo-responsive polymers discussed, the heteroatom—and not the carboxyl moiety—anchors the hydrophilic side chains to the backbone in the case of PNVCL.

For labeling PNVCL by fluorophores, copolymerization of **NVCL** with dye-functionalized comonomers is advantageous [13,15], as both the monomer **NVCL** and the homopolymer are difficult to functionalize. However, **NVCL** is much less reactive in radical copolymerization than styrenic and (meth)-acrylic derivatives. We therefore designed a series of new solvatochromic naphthalimide-bearing comonomers derived from maleic acid (Figure 1), in order to ensure the incorporation of isolated fluorophores into the polymers and thus, to exclude possible local clustering effects on the spectral properties. Although maleic acid derivatives do not homopolymerize, they are known to copolymerize reasonably well with *N*-vinylamides, with reactivity ratios close to 0 [61,62,63,64]. Moreover, as only one of the two carboxyl groups is needed to anchor the fluorophore to the polymerizable moiety, the second carboxyl group can be used for incorporating additional functional groups. We took advantage of this option by introducing substituents of diverse polarity, *i.e.*, ranging from highly hydrophilic to strongly hydrophobic, in the near vicinity of the fluorophore within an identical thermo-sensitive polymer matrix. In this way, we aimed at exploring the influence of the local molecular environment on the spectral properties of the dye label, below as well as above the coil-to-globule phase transition.

## 2. Experimental Section

### 2.1. Materials

#### 2.1.1. General

Chemicals: acetone (Carl Roth GmbH, Karlsruhe, Germany, ≥99.5%, ), 1-adamantanemethylamine (Sigma Aldrich Chemie GmbH, Taufkirchen, Germany, 98%), aminoethanol (Sigma Aldrich, ≥99%), aminoethylpiperazine (Sigma Aldrich, 99%), *O*-(7-azabenzotriazol-1-yl)-*N*,*N*,*N*′,*N*′‑tetramethyluronium hexafluorophosphate (HATU, Chempur, Karlsruhe, Germany, >95%), *n*-butyl-1-amine (Sigma Aldrich, 99.5%), chloroform (Roth, ≥99%), 4‑chloro-1,8-naphthalic anhydride (Alfa Aesar, Karlsruhe, Germany, 94%), dichloromethane (Merck Schuchard, Hohenbrunn, Germany, dry, max. 0.004% H_2_O), dichloromethane (Roth, ≥99.5%), diethyl ether (Th. Geyer, Renningen, Germany, 99.5%), *N*,*N*‑diisopropylethylamine (DIEA, Sigma Aldrich, >99%), ethanol (abs. pure, Chemsolute, Th. Geyer, Renningen, Germany, 99.5%), hydrochloric acid (1 M, Chemsolute), maleic anhydride (Sigma Aldrich, 95%), methanol (Sigma Aldrich, anhydrous, 99.8%), methoxyethanol (ABCR GmbH, Karlsruhe, Germany, 98%), morpholine (Alfa Aesar, 99%), octadecylamine (Sigma Aldrich, 97%), piperidine (Sigma Aldrich, 99%), propargylamine (Sigma Aldrich, 98%), 1,2,3,4-tetra-*O*-acetyl-ß-d-glucopyranose (Carbosynth), triethylamine (pure, Acros Organics, Fisher Scientific GmbH, SchwerteGermany, 99.7%), and *N*‑vinyl-caprolactam (NVCL, Sigma Aldrich, 98%), were used as received. Tris(triphenylphosphine)copper(I) bromide was a gift from Sandor Dippel (University of Potsdam, Potsdam, Germany). 2,2′-Azobisisobutyronnitrile (AIBN, Sigma Aldrich, 98%) and 4,4′-azobis-(4-cyanopentanecarboxylic acid) (V‑501, Wako Chemicals GmbH, Neuss, Germany) were crystallized from methanol before use. Tetraacetyl-1-ethoxy-2-azido-mannose was synthesized in a three step process following a literature procedure [65,66].

#### 2.1.2. Synthesis of Intermediates

##### 2-(2-hydroxyethyl)-6-morpholino-benzo[de]isoquinoline-1,3-dione (**2**)

Anhydride 6-(morpholino)-benzo[de]isochromene-1,3-dione was synthesized following a literature procedure [67], yield 74%. Mass spectrum (HRMS-EI): signal at 283.0833 [M]^+^ (calcd: 283.0845). ^1^H NMR (300 MHz, CDCl_3_, δ in ppm) δ 8.54 (d, *J* = 7.3 Hz, 1H, H_aryl_), 8.49 (d, *J* = 8.2 Hz, 1H, H_aryl_), 8.46 (d, *J* = 7.9 Hz, 1H, H_aryl_), 7.73 (t, *J* = 7.9 Hz, 1H, H_aryl_), 7.24 (d, *J* = 8.2 Hz, 1H, H_aryl_), 4.03 (t, *J* = 4.5 Hz, 4H, H_C*H2*–O–C*H2*_), 3.32 (t, *J* = 4.5 Hz, 4H, H_C*H2*–N–aryl_). ^13^C NMR (APT, 75 MHz, CDCl_3_, δ in ppm) δ 161.22 (+), 160.51 (+), 156.99 (+), 134.96 (−), 133.39 (−), 132.38 (+), 131.72 (−), 126.29 (−), 119.67 (+), 115.40 (−), 113.76 (−), 112.55 (+), 66.97 (+), 53.46 (+). IR (cm^−1^, selected bands): 2959 *ν*(CH_3_), 2853 *ν*(CH_2_), 1770 *ν*(C=O), 1727 *ν*(C=O), 1574 *ν*(C=C_arom._). The anhydride (2.00 g, 7.06 mmol, 1 eq.) and 2-amino-ethanol (0.06 g, 9.88 mmol, 0.59 mL, 1.4 eq.) in 30 mL of ethanol were refluxed for 3 h. After evaporating the solvent, the residue was dissolved in dichloromethane and extracted thrice with water. The organic phase was dried over sodium sulfate, the solvent was evaporated, and the remaining solid was dried *in vacuo*. Yield: 1.67 g (5.12 mmol, 73%). Mass spectrum (HRMS-EI): signal at 326.1257 [M]^+^ (calcd: 326.1267).

^1^H NMR (300 MHz, CDCl_3_, δ in ppm) δ 8.57 (dd, *J* = 7.3, 0.8 Hz, 1H, H_aryl_), 8.51 (d, *J* = 8.1 Hz, 1H, H_aryl_), 8.41 (dd, *J* = 8.4, 0.8 Hz, 1H, H_aryl_), 7.69 (dd, *J* = 8.3, 7.5 Hz, 1H, H_aryl_), 7.21 (d, *J* = 8.1 Hz, 1H, H_aryl_), 4.43 (t, *J* = 5.3 Hz, 2H, H_C*H2*–N–C=O_), 4.05–3.92 (m, 6H, H_C*H2*–O–C*H2*_ + H_C*H2*–OH_), 3.26 (t, *J* = 4.6 Hz, 4H, H_C*H2*–N–aryl_), 2.62 (t, *J* = 5.2 Hz, 1H, H_CH2–CH2–O*H*_).

^13^C NMR (APT, 75 MHz, CDCl_3_, %. δ in ppm) δ 165.28 (+), 164.86 (+), 156.00 (+), 132.93 (−), 131.52 (−), 130.43 (−), 130.04 (+), 126.17 (+), 125.93 (−), 123.15 (+), 116.87 (+), 115.06 (−), 67.04 (+), 61.93 (+), 53.55 (+), 42.82 (+).

IR (cm^−1^, selected bands): 2961 *ν*(C–H), 2854 *ν*(C–H), 1691 *ν*(C=O), 1647 *ν*(C=O), 1587 *ν*(C=C_arom._).

##### 6-morpholino-2-(2-(piperazin-1-yl)ethyl)-benzo[de]isoquinoline-1,3-dione (**3**)

6-(morpholino)-benzo[*de*]isochromene-1,3-dione (2.00 g, 7.06 mmol, 1 eq.) and 2-amino-ethylpiperazine (1.28 g, 1.30 mL, 9.88 mmol, 1,4 eq.) in 30 mL of ethanol were refluxed for 16 h. After evaporating the solvent, the residue is dissolved in chloroform and extracted thrice with water. The organic phase was dried over sodium sulfate, the solvent was evaporated, and the remaining solid was dried *in vacuo*. Yield: 2.58 g (6.54 mmol, 93%).

Mass spectrum (HRMS-EI): signal at 394.2006 [M]^+^ (calcd: 394.2005).

^1^H NMR (300 MHz, CDCl_3_, δ in ppm) δ 8.54 (dd, *J* = 7.3, 1.1 Hz, 1H, H_aryl_), 8.49 (d, *J* = 8.1 Hz, 1H, H_aryl_), 8.39 (dd, *J* = 8.5, 1.1 Hz, 1H, H_aryl_), 7.67 (dd, *J* = 15.8, 1.1 Hz, 1H, H_aryl_), 7.20 (d, *J* = 8.1 Hz, 1H, H_aryl_), 4.30 (t, *J* = 7.2 Hz, 2H, H_C*H2*–N–C=O_), 3.99 (m, *J* = 4.6 Hz, 4H, H_C*H2*–O–C*H2*_), 3.24 (t, *J* = 4.6 Hz, 4H, H_C*H2*–N–aryl_), 2.84 (t, *J* = 4.8 Hz, 4H, H_C*H2*–N(CH2)–C*H2*_), 2.65 (t, *J* = 7.2 Hz, 2H, H_O=CN C–C*H2*–N<_), 2.55 (m, 4H, H_C*H2*–NH–C*H2*_), 1.82 (s, 1H, H_N*H*_).

^13^C NMR (APT, 75 MHz, CDCl_3_, δ in ppm) δ 164.42 (+), 163.96 (+), 155.69 (+), 132.54 (−), 131.20 (−), 130.10 (−), 129.98 (+), 126.25 (+), 125.91 (−), 123.42 (+), 117.27 (+), 115.05 (−), 67.06 (+), 56.43 (+), 54.83 (+), 53.55 (+), 46.23 (+), 37.32 (+).

IR (cm^−1^, selected bands): 3563 *ν*(N–H), 2949 *ν*(C–H), 2823 *ν*(C–H), 1694 *ν*(C=O), 1652 *ν*(C=O), 1589 *ν*(C=C_arom._).

##### 4-(2-(6-morpholino-1,3-dioxo-benzo[de]isoquinolin-2-yl)ethoxy)-4-oxobut-2-enoic acid (**4**)

2-(2-hydroxyethyl)-6-morpholino-benzo[*de*]isoquinoline-1,3-dione (**2**) (1.00 g, 3.06 mmol, 1 eq.), maleic anhydride (0.90 g, 9.2 mmol, 3 eq.) and triethylamine (0.47 g, 0.64 mL, 4.6 mmol, 1.5 eq.) in 20 mL of acetone were stirred for 24 h at ambient temperature. After evaporating the solvent, the residue is dissolved in chloroform and extracted thrice with water. The organic phase was dried over sodium sulfate, the solvent was evaporated, and the remaining solid was dried *in vacuo*. Yield: 1.03 g (2.43 mmol, 79%).

Mass spectrum (HRMS-EI): signal at 424.1251 [M]^+^ (calcd: 424.1271).

^1^H NMR (300 MHz, CDCl_3_, δ in ppm) δ 8.59 (dd, *J* = 7.3, 0.8 Hz, 1H, H_aryl_), 8.53 (d, *J* = 8.1 Hz, 1H, H_aryl_), 8.45 (dd, *J* = 8.4, 0.8 Hz, 1H, H_aryl_), 7.72 (dd, *J* = 15.8, 0.8 Hz, 1H, H_aryl_), 7.23 (m, 1H, H_aryl_), 6.44 (d, *J* = 12.8 Hz, 1H, H_HOOC–C*H*=CH–COOCH2_), 6.28 (d, *J* = 12.8 Hz, 1H, H_HOOC–CH=C*H*–COOCH2_), 4.65 and 4.57 (m and m, 2+2H, H_CON–C*H2*–C*H2*–OOC_), 4.02 (t, *J* = 4.5 Hz, 4H, H_C*H2*–O–C*H2*_), 3.28 (t, *J* = 4.5 Hz, 4H, H_C*H2*–N–aryl_).

^13^C NMR (APT, 75 MHz, CDCl_3_, δ in ppm) δ 167.40 (+), 164.67 (+), 164.65 (+), 164.15 (+), 156.19 (+), 136.59 (−), 135.78 (−), 133.88 (−), 133.06 (−), 131.62 (−), 130.67 (−), 130.10 (+), 128.96 (−), 126.25 (+), 126.00 (−), 122.92 (+), 116.58 (+), 115.14 (−), 67.02 (+), 64.11 (+), 53.53 (+), 38.58 (+).

IR (cm^−1^, selected bands): 3469 *ν*(O–H), 2966 *ν*(C–H), 2853 *ν*(C–H), 1729 *ν*(C=O), 1694 *ν*(C=O), 1652 *ν*(C=O), 1586 *ν*(C=C_arom._).

##### 4-(4-(2-(6-morpholino-1,3-dioxo-benzo[de]isoquinolin-2-yl)ethyl)piperazin-1-yl)-4-oxobut-2-enoic acid (**5**)

6-morpholino-2-(2-(piperazin-1-yl)ethyl)-benzo[*de*]isoquinoline-1,3-dione (**3**) (2.00 g, 5.08 mmol, 1 eq.), maleic anhydride (0.74 g, 7.62 mmol, 1.5 eq.) and triethylamine (0.76 g, 1.06 mL, 7.62 mmol, 1.5 eq.) in 30 mL of acetone were stirred for 2 h at ambient temperature. The precipitate formed is filtered off, and dried *in vacuo*. Yield: 1.80 g (contains *ca.* 10 wt % of triethylamine, (3.3 mmol, 65%).

Mass spectrum (HRMS-ESI): signal at 493.2093 [M + H]^+^ (calcd: 493.2082).

^1^H NMR (300 MHz, CDCl_3_, δ in ppm) δ 8.55 (d, *J* = 7.2 Hz, 1H, H_aryl_), 8.49 (d, *J* = 8.1 Hz, 1H, H_aryl_), 8.40 (d, *J* = 8.4 Hz, 1H, H_aryl_), 7.69 (t, *J* = 7.9 Hz, 1H, H_aryl_), 7.22 (d, *J* = 8.1 Hz, 1H, H_aryl_), 6.26 (d, *J* = 12.1 Hz, 1H, H_OOC–C*H*=CH–CON_), 6.05 (d, *J* = 12.1 Hz, 1H, H_OOC–CH=C*H*–CON_), 4.31 (t, *J* = 6.7 Hz, 2H, H_C*H2*–N–(C=O)2_), 4.00 (t, *J* = 4.0 Hz, 4H, H_C*H2*–O–C*H2*_), 3.63 (unresolved t, 2H, H_C*H2*–N(C=O)‑CH2 trans_), 3.49 (unresolved t, 2H, H_CH2–N(C=O)–C*H2* cis_), 3.25 (t, *J* = 3.9 Hz, 4H, H_C*H2*–N–aryl_), 2.73 (t, *J* = 6.7 Hz, 2H, H_>N–C*H2*–CH2–N(C=O)2_), 2.62 (unresolved t, 4H, _HCH2–N(C*H)2*_).

^13^C NMR (APT, 75 MHz, CDCl_3_, δ in ppm) δ 170.21 (+), 167.63 (+), 164.49 (+), 164.02 (+), 155.82 (+), 132.64 (−), 131.95 (−), 131.28 (−), 130.24 (−), 130.06 (−), 126.32 (+), 125.97 (−), 123.41 (+), 117.23 (+), 115.11 (−), 67.08 (+), 55.77 (+), 53.59 (+), 53.24 (+), 52.81 (+), 45.23 (+), 37.43 (+).

IR (cm^−1^, selected bands): 2972 ν(C–H), 2820 ν(C–H), 1694 ν(C=O), 1651 ν(C=O), 1585 ν(C=C_arom._).

#### 2.1.3. Synthesis of Dye Monomers

Dye monomers (**1a**)–(**1d**) were synthesized by a general procedure, varying the functional amine, *i.e.*, *n*-octadecylamine, adamantylmethylamine, *n-*butylamine, or piperidine, to be coupled. In the standard recipe, DIEA (1.2 eq.) and the functional amine (1.1 eq.) were added to a solution of 4-(4-(2-(6-morpholino-1,3-dioxo-benzo[*de*]isoquinolin-2-yl)-ethyl)-piperazin-1-yl)-4-oxobut-2-enoic acid (**5**) (1 eq.) and HATU (1.2 eq.) in dry dichloromethane. The mixture was stirred for 16 h under argon atmosphere at ambient temperature. After adding chloroform, the mixture was extracted once with 1 M hydrochloric acid and thrice with water. The organic phase was dried over sodium sulfate, the solvent was evaporated, and the remaining solid was dried *in vacuo*.

##### 4-(4-(2-(6-morpholino-1,3-dioxo-benzo[de]isoquinolin-2-yl)ethyl)piperazin-1-yl)-*N*-octadecyl-4-oxobut-2-enamide (**1a**)

Coupling with *n*-octadecylamine, yield 27%.

Mass spectrum (HRMS-ESI): signal at 744.5059 [M + H]^+^ (calcd: 744.5058).

^1^H NMR (300 MHz, CDCl_3_, δ in ppm) δ 8.53 (d, *J* = 7.2 Hz, 1H, H_aryl_), 8.47 (d, *J* = 8.1 Hz, 1H, H_aryl_), 8.40 (d, *J* = 8.4 Hz, 1H, H_aryl_), 7.69 (t, *J* = 7.9 Hz, 1H, H_aryl_), 7.21 (d, *J* = 8.1 Hz, 1H, H_aryl_), 6.29 (d, *J* = 12.4 Hz, 1H, H_CH2HNOC–HC=C*H*–CON(CH2)2_), 6.05 (d, *J* = 12.4 Hz, 1H, H_CH2HNOC–*H*C=CH–CON(CH2)2_), 4.37 (t, *J* = 4.9 Hz, 2H, H_C*H2*–N(C=O)2_), 4.00 (unresolved t, 4H, H_C*H2*–O–C*H2*_), 3.67 (bs, 2H, H_C*H2*–N(C=O)–CH2 cis_), 3.53 (bs, 2H, H_CH2–N(C=O)–C*H2 trans*_), 3.26 (unresolved t, 4H, H_C*H2*N–aryl_), 3.17 (m, 2H, H_CONH–C*H2*_), 3.10–2.75 (m, 6H, H_C*H2*–N(C*H2*)2_), 1..46 (m, 2H, H_CONH–CH2–C*H2*_), 1.23–1.24 (m, 30H, H_(C*H2*)15–CH3_), 0.87 (t, *J* = 6.6 Hz, 3H, H_C*H3*_).

^13^C NMR (APT, 75 MHz, CDCl_3_, δ in ppm) δ 166.53 (+), 164.82 (+), 164.71 (+), 164.34 (+), 156.22 (+), 133.00 (−), 131.56 (−), 130.69 (−), 130.47 (−), 130.33 (+), 130.13 (+), 126.25 (+), 126.02 (−), 123.06 (+), 116.70 (+), 115.17 (−), 67.09 (+), 56.17 (+), 53.62 (+), 52.93 (+), 52.65 (+), 45.45 (+), 40.41 (+), 39.87 (+), 36.35 (+), 32.07 (+), 29.86 (+), 29.80 (+), 29.75 (+), 29.50 (+), 29.48 (+), 29.41 (+), 27.14 (+), 22.83 (+), 14.23 (−).

IR (cm^−1^, selected bands): 3413 *ν*(N–H), 3303 *ν*(N–H), 2922 *ν*(C–H), 2852 *ν*(C–H), 1693 *ν*(C=O), 1656 *ν*(C=O), 1588 *ν*(C=C_arom._).

##### *N*-((-adamantan-1-yl)methyl)-4-(4-(2-(6-morpholino-1,3-dioxo-benzo[de]iso-quinolin-2-yl)ethyl)piperazin-1-yl)-4-oxobut-2-enamide (**1b**)

Coupling with adamantylmethylamine, yield nearly quantitative (contaminated by DIEHA).

Mass spectrum (HRMS-EI): signal at 639.3423 [M]^+^ (calcd: 639.3415).

^1^H NMR (300 MHz, CDCl_3_, δ in ppm) δ 8.54 (d, *J* = 7.1 Hz, 1H, H_aryl_), 8.48 (d, *J* = 8.1 Hz, 1H, H_aryl_), 8.41 (d, *J* = 8.3 Hz, 1H, H_aryl_), 7.70 (t, *J* = 7.8 Hz, 1H, H_aryl_), 7.22 (d, *J* = 8.1 Hz, 1H, H_aryl_), 6.32 (d, *J* = 12.5 Hz, 1H, H_NOC–HC=C*H*–CON–CH2–CH2-N_), 6.10 (d, *J* = 12.5 Hz, 1H, H_NOC–*H*C=CH–CON–CH2–CH2–N_), 4.38 (t, *J* = 5.5 Hz, 2H, H_C*H2*–N(C=O)2_), 4.01 (unresolved t, 4H, H_C*H2*–O–C*H2*_), 3.70 (m, 2H, H_C*H2*–N(C=O)–CH2 cis_), 3.55 (m, 2H, H_CH–N(C=O–C*H2* trans_), 3.27 (unresolved t, 4H, H_C*H2*–N–aryl_), 2.92 (m, 9H, 4‑H_C*H*–N(CH2–C*H2*_ + 2‑H_CH–C*H*–N(CH2)2_ + 2‑H_H–C*H*–CR3 (**1′**)_ + H_N*H*_), 1.95 (s, 3H, H_CH–Adamantan_), 1.68–1.35 (m, 12H, H_CH2–adamantan_).

^13^C NMR (APT, 75 MHz, CDCl_3_, δ in ppm) δ 164.77 (+), 164.70 (+), 164.17 (+), 162.41 (+), 156.13 (+), 132.93 (−), 131.52 (−), 130.60 (−), 130.19 (+), 126.33 (+), 126.00 (−), 123.19 (+), 116.90 (+), 115.16 (−), 67.08 (+), 56.94 (+), 55.44 (+), 53.60 (+), 51.33 (+), 40.31 (+), 40.21 (+), 37.03 (+), 36.77 (+), 34.48 (+), 33.74 (+), 28.36 (−).

IR (cm^−1^, selected bands): 3326 *ν*(N–H), 2900 *ν*(C–H), 2846 *ν*(C–H), 1694 *ν*(C=O), 1653 *ν*(C=O), 1613 *ν*(C=O), 1587 *ν*(C=C_arom._).

##### *N*-butyl-4-(4-(2-(6-morpholino-1,3-dioxo-benzo[de]isoquinolin-2-yl)ethyl)piperazin-1-yl)-4-oxobut-2-enamide (**1c**)

Coupling with *n*-butylamine, yield *ca.* 70% (contaminated by tetramethylurea and DIEA).

Mass spectrum (HRMS-EI): signal at 547.2700 [M]^+^ (calcd: 547.2789).

^1^H NMR (300 MHz, CDCl_3_, δ in ppm) δ 8.56 (dd, *J* = 7.3, 1.0 Hz, 1H, H_aryl_), 8.51 (d, *J* = 8.1 Hz, 1H, H_aryl_), 8.42 (dd, *J* = 8.4, 0.9 Hz, 1H, H_aryl_), 7.70 (dd, *J* = 7.4, 0,96 Hz, 2H, 1-H_aryl_ + 1-H_N*H*_), 7.23 (d, *J* = 8.1 Hz, 1H, H_aryl_), 6.31 (d, *J* = 12.9 Hz, 1H, H_NOC–HC=C*H*–CO–N–CH2–CH2–N_), 6.07 (d, *J* = 12.9 Hz, 1H, H_NOC–*H*C=CH–CON–CH2–CH2–N_), 4.31 (t, *J* = 6.6 Hz, 2H, H_C*H2*–N(C=O)2_), 4.01 (t, *J* = 4.5 Hz, 4H, H_C*H2*–O–C*H2*_), 3.61 (m, 2H, H_–C*H2*–N(C=O)–CH2 cis_), 3.42 (m, 2H, H*_CH2_*_–N(C=O)–CH2 trans_), 3.30–3.21 (m, 6H, H_aryl–N(C*H2)2*_ + H_CONH–C*H2*–)_), 2.72 (t, *J* = 6.6 Hz, 2H, H_NC*H2*–CH2–N(C=O)2_), 2.62–2.52 (m, 4H, H_>N(C*H2)2*_), 1.48 (m, 2H, H_C*H2*–C–NHCO–_), 1.33 (m, 2H, H_C*H2*–CH3_), 0.89 (t, *J* = 7.3 Hz, 3H, H_C*H3*_).

^13^C NMR (APT, 75 MHz, CDCl_3_, δ in ppm) δ 165.85 (+), 164.94 (+), 164.63 (+), 164.15 (+), 155.93 (+), 132.74 (−), 131.75 (−), 131.39 (−), 130.33 (−), 130.16 (+), 129.05 (−), 126.43 (+), 126.07 (−), 123.50 (+), 117.32 (+), 115.22 (−), 67.17 (+), 55.76 (+), 53.68 (+), 53.38 (+), 52.97 (+), 46.82 (+), 41.87 (+), 39.45 (+), 37.45 (+), 31.62 (+), 20.27 (+), 13.92 (−).

IR (cm^−1^, selected bands): 3293 *ν*(N–H), 3070 *ν*(C–H), 2959 *ν*(C–H), 2925 *ν*(C–H), 2357 *ν*(C–H_arom._), 1693 *ν*(C=O), 1651 *ν*(C=O), 1614 *ν*(C=O), 1588 *ν*(C=C_arom._).

##### 6-morpholino-2-(2-(4-(4-oxo-4-(piperidin-1-yl)but-2-enoyl)piperazin-1-yl)ethyl)-benzo[de]isoquinoline-1,3-dione (**1d**)

Coupling with piperidine, yield *ca.* 35% (contaminated by tetramethylurea and DIEA).

Mass spectrum (HRMS-ESI): signal at 560.2848 [M + H]^+^ (calcd: 560.2867).

^1^H NMR (300 MHz, CDCl_3_, δ in ppm) δ 8.59 (d, *J* = 7.2 Hz, 1H, H_aryl_), 8.53 (d, *J* = 8.2 Hz, 1H, H_aryl_), 8.44 (d, *J* = 8.4 Hz, 1H, H_aryl_), 7.73 (t, *J* = 7.9 Hz, 1H, H_aryl_), 7.25 (d, *J* = 8.2 Hz, 1H, H_aryl_), 6.42–6.22 (m, *J* = 12.1 Hz, 2H, H_>NCO–C*H*=C*H*–CON<_), 4.34 (t, *J* = 6.5 Hz, 2H, H_C*H2*–N(CO)2_), 4.04 (unresolved t, 4H, H_C*H2*–O–C*H2*_), 3.61–3.43 (m, 8H, 2 × H_CON(C*H2*)2_), 3.29 (unresolved t, 4H, H_C*H2*N–aryl_), 2.74 (t, *J* = 6.4 Hz, 2H, H_C*H2*–N<_), 2.61–2.60 (m, 4H, H_–N_(_C*H2*)2)–_), 1.61–1.58 (m, 6H, H_–_(_C*H2*)3–_).

^13^C NMR (75 MHz, CDCl_3_, δ in ppm) δ 165.59 (+), 165.55 (+), 164.78 (+), 164.31 (+), 156.04 (+), 132.89 (−), 131.55 (−), 130.45 (−), 130.32 (+), 129.98 (−), 128.36 (−), 126.60 (+), 126.23 (−), 123.70 (+), 117.55 (+), 115.38 (−), 67.36 (+), 66.20, 55.95 (+), 53.85 (+), 53.58 (+), 53.22 (+), 47.83 (+), 46.79 (+), 42.73 (+), 41.89 (+), 37.65 (+), 26.60 (+), 25.76 (+), 24.90 (+).

IR (cm^−1^, selected bands): 2933 *ν*(C–H), 2854 *ν*(C–H), 1693 *ν*(C=O), 1650 *ν*(C=O), 1624 *ν*(C=O), 1587 *ν*(C=C_arom._).

##### 2-(acetoxymethyl)-6-(2-(4-((-4-(4-(2-(6-morpholino-1,3-dioxo-benzo[de]isoquinolin-2-yl)ethyl)piperazin-1-yl)-4-oxobut-2-enamido)methyl)-1,2,3-triazol-1-yl)ethoxy)tetrahydro-pyran-3,4,5-triyl triacetate (**1e**)

Monomer (**1e**) is synthesized in two steps. In the first step, the synthetic procedure of monomers **1a**–**d** is followed using propargylamine as functional amine to be coupled, yield 90%.

Mass spectrum (HRMS-ESI): signal at 530.2414 [M + H]^+^ (calcd: 530.2398). ^1^H NMR (300 MHz, CDCl_3_) δ 8.57 (dd, *J* = 7.3, 1.0 Hz, 1H, H_aryl_), 8.52 (d, *J* = 8.1 Hz, 1H, H_aryl_), 8.43 (dd, *J* = 8.4, 1.0 Hz, 1H, H_aryl_), 8.17 (s, 1H, H_N*H*_), 7.71 (dd, *J* = 8.4, 1.0 Hz, 1H, H_aryl_), 7.24 (d, *J* = 8.1 Hz, 1H, H_aryl_), 6.39 (d, *J* = 12.9 Hz, 1H, H_NOC–HC=C*H*–CON_), 6.09 (d, *J* = 12.9 Hz, 1H, H_HNOC–*H*C=CH–CON_), 4.32 (t, *J* = 6.6 Hz, 2H, H_C*H2*–N–(C=O)2_), 4.11–3.93 (m, 6H, H_C*H2*–O–C*H2*_ + H_C*H2*–C ≡C_), 3.63 (t, *J* = 4.8 Hz, 2H, H_C*H2*–N(C=O)–CH2 cis_), 3.48 (t, *J* = 4.8 Hz, 2H, H_CH2–N(C=O)–C*H2* trans_), 3.27 (t, *J* = 4.5 Hz, 4H, H_C*H2*–N–aryl_), 2.73 (t, *J* = 6.6 Hz, 2H, H_C*H2*–N<_), 2.62–2.57 (m, 4H, H_C*H2*–N(CH2)–C*H2*_), 2.20 (t, *J* = 2.5 Hz, 1H, H_C ≡C*H*_).

^13^C NMR (APT, 75 MHz, CDCl_3_) δ 165.45 (+), 164.57 (+), 164.53 (+), 164.10 (+), 155.88 (+), 132.70 (−), 131.34 (−), 131.07 (−), 130.29 (−), 130.10 (+), 129.85 (−), 126.37 (+), 126.02 (−), 123.43 (+), 117.24 (+), 115.16 (−), 71.53 (+), 67.11 (+), 55.66 (+), 53.62 (+), 53.27 (+), 52.87 (+), 46.80 (+), 41.94 (+), 37.38 (+), 29.24 (+).

IR (cm^−1^, selected bands): 3257 *ν*(N–H), 2969 *ν*(C–H), 2901 *ν*(C–H), 2110 *ν*(C≡C), 1692 *ν*(C=O), 1648 *ν*(C=O), 1614 *ν*(C=O), 1588 *ν*(C=C_arom._).

In the second step, the propargylamide intermediate (1 eq.), DIEA (3 eq.) and tetraacetyl-1-ethoxy-2-azido-mannose (1 eq.) in 1 mL of dry CH_2_Cl_2_ were purged for 10 min with argon. After adding bromo‑tris(triphenylphosphine)copper(I) (0.1 eq.), the mixture was stirred for 24 h at 40 °C. The solvent was evaporated, the residue dissolved in little methanol, and the mixture precipitated by adding a large excess of diethylether. The product is filtered off and dried *in vacuo*. Yield 72%, yellow solid.

Mass spectrum (HRMS-ESI): signal at 947.3740 [M + H]^+^ (calcd: 947.3781).

^1^H NMR (300 MHz, CDCl_3_) δ 8.57 (d, *J* = 7.1 Hz, 1H, H_aryl_), 8.51 (d, *J* = 8.2 Hz, 1H, H_aryl_), 8.42 (d, *J* = 8.3 Hz, 1H, H_aryl_), 7.89 (s, 1H, H_N*H*_), 7.73–7.66 (m, 2H, H_aryl_ + H_triazol_), 7.23 (d, *J* = 8.2 Hz, 1H, H_aryl_), 6.34 (d, *J* = 12.2 Hz, 1H, H_HNOC–HC=C*H*–CON(CH2)2_), 6.09 (d, *J* = 12.2 Hz, 1H, H_HNOC–*H*C=CH–CON(CH2)2_), 5.29–5.07 (m, 3H, H_C*H*–OAc_), 4.75 (s, 1H, H_O–C*H*–O–Mannose_), 4.57 (broad s, 4H, H_CH –triazol–C*H2*_), 4.31 (t, *J* = 6.4 Hz, 2H, H_C*H2*–N(C=O)2_), 4.19–3.95 (m, 7H, H_C*H2*OAc_ + H_C*H*–O–C–O_ + H_C*H2*–O–C*H2*_), 3.91–3.82 (m, 1H, H_O–HC*H*–CH2-triazol_), 3.61 (unresolved t, 2H, H_C*H2*–N(C=O)–CH2 cis_), 3.52–3.35 (m, 3H, H_CH2–N(C=O)–C*H2* trasns_ +1-H_O–*H*CH–CH2-triazol_), 3.26 (t, *J* = 4.0 Hz, 4H, H_C*H2*N–aryl_), 2.72 (t, *J* = 6.4 Hz, 2H, H_C*H2*–N(CH2)2_), 2.61 (m, H_C*H2*–N(CH2)–C*H2*_, 2.12 (s, 3H, H_acetyl_), 2.09 (s, 3H, H_acetyl_), 2.04 (s, 3H, H_acetyl_), 1.99 (s, 3H, H_acetyl_).

^13^C NMR (APT, 75 MHz, CDCl_3_) δ 170.15 (+), 170.13 (+), 169.85 (+), 169.83 (+), 165.78 (+), 164.72 (+), 164.69 (+), 164.11 (+), 155.86 (+), 145.09 (+), 132.70 (−), 132.33 (−), 132.30 (−), 132.07 (−), 132.03 (+), 131.35 (−), 130.28 (−), 130.11 (+), 129.46 (−), 128.72 (+), 128.56 (−), 126.38 (−), 126.03 (−), 117.29 (+), 115.17 (−), 97.54 (−), 69.43 (−), 69.23 (−), 68.98 (−), 67.13 (+), 66.29 (+), 65.92 (−), 62.41 (+), 55.71 (+), 53.63 (+), 53.16 (+), 52.89 (+), 49.82 (+), 46.64 (+), 41.72 (+), 37.43 (+), 35.39 (+), 20.96 (−), 20.89 (−), 20.84 (−).

IR (cm^−1^, selected bands): 3347 ν(N∓H), 2923 ν(C∓H), 2853 ν(C∓H), 1744 ν(C=O), 1694 ν(C=O), 1653 ν(C=O), 1589 ν(C=C_arom._).

##### 6-(((-4-(4-(2-(6-morpholino-1,3-dioxo-benzo[de]isoquinolin-2-yl)ethyl)piperazin-1-yl)-4-oxobut-2-enoyl)oxy)methyl)tetrahydro-pyran-2,3,4,5-tetrayl tetraacetate (**1f**)

DIEA (1.2 eq.) and 1,2,3,4-tetra-*O*-acetyl-ß-D-glucopyranose (0.07 g, 0.20 mmol, 1.1 eq.) were added to a solution of 4-(4-(2-(6-morpholino-1,3-dioxo-benzo[*de*]isoquinolin-2-yl)-ethyl)- piperazin-1-yl)-4-oxobut-2-enoic acid (**5**) (1 eq.) and HATU (1.2 eq.) in dry dichloromethane. The mixture was stirred for 16 h under argon atmosphere at ambient temperature. The solvent was evaporated, and the residue was purified by column chromatography on silica gel (eluent CHCl_3_:CH_3_OH 30:1). Yield *ca.* 47%.

Mass spectrum (HRMS-ESI): signal at 823.3070 [M + H]^+^ (calcd: 823.3032).

^1^H NMR (300 MHz, CDCl_3_, δ in ppm) δ 8.57 (dd, *J* = 7.3, 1.0 Hz, 1H, H_aryl_), 8.51 (d, *J* = 8.2 Hz, 1H, H_aryl_), 8.43 (dd, *J* = 8.4, 1.0 Hz, 1H, H_aryl_), 7.71 (dd, *J* = 7.4, 1.0 Hz, 1H, H_aryl_), 7.24 (d, *J* = 8.2 Hz, 1H, H_aryl_), 6.56 (d, *J* = 11.9 Hz, 1H, H_OOC–HC=C*H*–CON_), 6.04 (d, *J* = 11.9 Hz, 1H, H_OOC–*H*C=CH–CON_), 5.71 (d, *J* = 8.1 Hz, 1H, H_glucose_), 5.28–5.06 (m, 3H, H_glucose_), 4.31 (t, *J* = 6.7 Hz, 2H, H_C*H2*–N(CO)2_), 4.24–4.17 (m, 2H, H_glucose_), 4.01 (unresolved t, 4H, H_C*H2*–O–C*H2*_), 3.87–3.84 (m, 1H, H_glucose_), 3.64 (m, 2H, H_C*H2*–N(C=O)–CH2 cis_), 3.36 (m, 2H, H_CH2–N(C=O)–C*H2* trans_), 3.26 (unresolved t, 4H, H_C*H2*N–aryl_), 2.72 (t, *J* = 6.7 Hz, 2H, H_N–CH2–C*H2*–N<_), 2.58 (m, 4H, H_N(C*H2*)2_), 2.10 (s, 3H, H_acetyl_), 2.02–2.00 (m, 9H, H_acetyl_).

^13^C NMR (APT, 75 MHz, CDCl_3_, δ in ppm) δ 170.16 (+), 169.41 (+), 169.30 (+), 168.99 (+), 165.20 (+), 164.53 (+), 164.10 (+), 164.06 (+), 155.83 (+), 139.19 (−), 132.65 (−), 131.30 (−), 130.24 (−), 130.08 (+), 126.35 (+), 125.99 (−), 123.44 (+), 122.38 (−), 117.27 (+), 115.14 (−), 91.81 (−), 72.93 (−), 72.75 (−), 70.41 (−), 68.10 (−), 67.10 (+), 62.22 (+), 55.75 (+), 53.61 (+), 53.08 (+), 52.77 (+), 46.31 (+), 41.40 (+), 37.43 (+), 20.90 (−), 20.71 (−), 20.67 (−), 20.65 (−).

IR (cm^−1^, selected bands): 2929 *ν*(C–H), 2856 *ν*(C–H), 1756 *ν*(C=O), 1694 *ν*(C=O), 1649 *ν*(C=O), 1588 *ν*(C=C_arom._).

##### 2-(6-morpholino-1,3-dioxo-benzo[de]isoquinolin-2-yl)ethyl-4-(((-adamantan-1-yl)-methyl)-amino)- 4-oxobut-2-enoate (**1g**)

DIEA (0.03 g, 0.05 mL, 0.26 mmol, 1.1 eq.) and 1-adamantyl-methylamine (0.04 g, 0.04 mL, 0.24 mmol, 1 eq.) are added to a solution of crude 4-(2-(6-morpholino-1,3-dioxo-benzo[*de*]iso- quinolin-2-yl)ethoxy)-4-oxobut-2-enoic acid (**4**) (0.10 g, 0.24 mmol, 1 eq.) and HATU (0.10 g, 0.26 mmol, 1.1 eq.) in dry CH_2_Cl_2_. The mixture is stirred for 16 h under argon atmosphere at ambient temperature. After adding chloroform, the mixture was extracted with 1 M HCl and thrice with water. The organic phase was dried over sodium sulfate, the solvent was evaporated, and the remaining solid was dried *in vacuo*. Yield *ca.* 65% (contains *ca.* 10 wt % of tetramethylurea, 0.16 mmol).

Mass spectrum (HRMS‑EI): signal at 571.2677 [M]^+^ (calcd: 571.2677).

^1^H NMR (300 MHz, CDCl_3_, δ in ppm) δ 8.58 (d, *J* = 7.2 Hz, 1H, H_aryl_), 8.52 (d, *J* = 8.1 Hz, 1H, H_aryl_), 8.43 (d, *J* = 8.4 Hz, 1H, H_aryl_), 8.34 (s, 1H, H_aryl_), 7.71 (t, *J* = 7.9 Hz, 1H, H_aryl_), 7.23 (d, *J* = 8.1 Hz, 1H, H_aryl_), 6.30 (d, *J* = 13.1 Hz, 1H, H_NOC–HC=C*H*–COO_), 6.01 (d, *J* = 13.1 Hz, 1H, H_NOC–*H*C=CH–COO_), 4.53 (bs, 4H, H_CON–C*H2*–C*H2*–OOC_), 4.01 (t, *J* = 4.4 Hz, 4H, H_C*H2*–O–C*H2*_), 3.27 (t, *J* = 4.4 Hz, 4H, H_C*H2*–N–aryl_), 2.96 (d, *J* = 6.0 Hz, 2H, H_adamantyl–C*H2*–NHCO_), 1.93 (s, 3H, H*_CH_*_–adamantane_), 1.7–1.35 (m, 12H, H*_CH2_*_–adamantane_).

^13^C NMR (APT, 75 MHz, CDCl_3_, δ in ppm) δ 166.11 (+), 164.66 (+), 164.44 (+), 164.14 (+), 156.10 (+), 139.53 (−), 132.94 (−), 131.55 (−), 130.53 (−), 130.18 (+), 126.37 (+), 126.04 (−), 124.11 (−), 123.17 (+), 116.90 (+), 115.17 (−), 67.08 (+), 62.90 (+), 53.61 (+), 51.58 (+), 40.27 (+), 38.87 (+), 37.10 (+), 33.81 (+), 28.45 (−).

IR (cm^−1^, selected bands): 3307 *ν*(N–H), 2901 *ν*(C–H), 2847 *ν*(C–H), 1697 *ν*(C=O), 1654 *ν*(C=O), 1588 *ν*(C=C_arom._).

#### 2.1.4. Synthesis of the Polymers

*N*-vinylcaprolactam (NVCL, 1 eq.), the selected dye-functionalized monomer (0.1 mol %) and azoinitiator (1 mol %) in dry methanol (25 w % solid content) were placed into a Schlenk flask equipped with a rubber septum. The mixture was purged with argon for 15 min and heated to 60 °C for 24 h in an oil bath. Then, the solution was cooled to ambient temperature, and dialyzed for 2 days against 50 vol % aqueous ethanol, and for 5 more days against water. The polymers containing dye comonomers **1e** and **1f** with the protected mannose or, respectively, glucose derived substituents were additionally dialyzed in semi-dilute aqueous K_2_CO_3_ (12.5 g·L^−1^) to split off the acetyl groups. The polymers were lyophilized to yield pale yellow powders. Table 1 lists the composition of the polymerization feeds and the yields of the dye-functionalized PNVCL samples prepared.

### 2.2. Methods

NMR-spectra were taken with a Bruker Avance 300 NMR spectrometer (Bruker, Billerica, MA, USA). ^1^H NMR spectra were recorded at 300 MHz, and ^13^C NMR spectra at 75 MHz (APT modus). The chemical shift δ was calibrated to the solvent signal. High resolution mass spectra (HRMS) were taken with a GC-MS Trace DSQ II (EI method), or an ESI-Q-TOFmicro mass spectrometer (ESI method) (both mass spectrometers: Thermo Scientific, Waltham, MA, USA). For samples taken with the EI method the relative mass *m*/*z* is given. For samples recorded with the ESI method, the mass is given plus the detected Ion (H^+^). IR spectra (ATR modus) were recorded with a NEXUS‑FT‑IR spectrometer with an ATR Smart Endurance Element (Thermo Instrument Systems Inc., Waltham, MA, USA). Size exclusion chromatography (SEC) was performed in dimethylformamide (DMF) as eluent. The set-up consists of a single-channel degasser (WGE Dr. Bures, Dallgow-Doeberitz, Germany), an isocratic pump P 1000 (Spectra Physics, Santa Clara, CA, USA), a set of columns (Guard 7,5 · 75 mm, PolarGel L 7,5 · 300 mm; Polymer Laboratories- Agilent Technologies, Santa Clara, California, USA), a UV‑VIS detector SEC 3010 and a refractometer SEC 3010 (WGE Dr. Bures, Dallgow-Döberitz, Germany). Measurements were taken at 50 °C with a flow rate of 1 mL·min^−1^, and calibrated with linear polystyrene standards (Polymer Standards Service; PSS, Mainz, Germany). UV-VIS signals were recorded at 398 nm for naphthalimide-bearing polymers. Absorption spectra in solution were recorded with a UV/VIS/NIR two beam spectrometer (Lamda 19, Perkin Elmer, Waltham, MA, USA, 1 nm slit, scan speed of 120 nm·min^−1^) in PMMA cuvettes (path length 10 mm). For turbidity studies, the transmittance of polymer solutions (3.00 g·L^−1^) was monitored by a photometer (Varian, Cary 50 UV-VIS, Palo Alto, CA, USA) at 600 nm, as function of temperature with heating and cooling rates of 0.2 K·min^−1^. Steady state fluorescence of polymer solutions was recorded a LS 50B Luminescence Spectrometer (Perkin Elmer, Waltham, MA, USA) in PMMA cuvettes (10 mm path length). Temperature dependent steady state fluorescence was measured with a Spectrofluorometer Jasco FP-8500 (Jasco International, Tokyo, Japan). The cuvette holder was equipped with a thermoelectric Peltier element for temperature control. Solutions were permanently stirred during the measurements with an external magnetic stirrer. Photoluminescence quantum yields were determined with a set-up Hamamatsu C9920-02 (Hamamatsu Photonics, Hamamatsu, Japan) that includes an integrating sphere with a photon multi-channel analyzer. The absorbance of the solutions was always kept below 0.1.

## 3. Results and Discussion

### 3.1. Synthesis of the Fluorophore-Labeled Polymers

**Figure 2:**
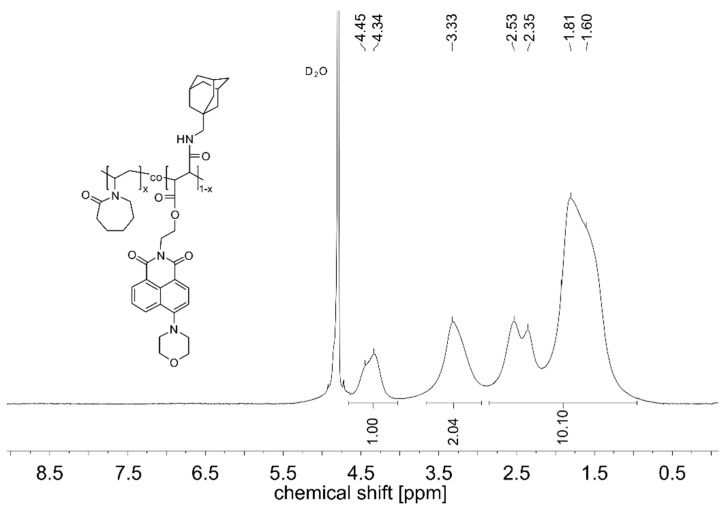
^1^H-NMR spectrum of copolymer **PNVCL-g** in D_2_O at ambient temperature.

The synthesis of the monomers followed classical strategies, using the dye-functionalized maleic acid monoester or monoamide, respectively, as intermediates. This enabled the facile and versatile variation of the second carboxyl substituent of the polymerizable maleic acid motif in the final step. The various new dye-functionalized maleamides or maleic esteramides were engaged as minority component in the radical copolymerization with NVCL in methanol. The relative amount of the dye comonomers was kept as low as 0.1 mol % in the reaction mixtures, to obtain polymers that incorporate the fluorophores in the midst of the chains, but do not contain more than one fluorophore per chain. Synthetic procedures and the analytical data of the copolymers obtained are summarized in Table 1. As the amounts of dye comonomer incorporated were too low to be analyzed by ^1^H NMR spectroscopy (see Figure 2), the amounts were derived from the visual absorbance band of the chromophore, assuming that the extinction coefficients of the monomers do not change upon incorporation into the polymers. Within the precision of the analysis, the copolymers contain the same amount of dye monomers as engaged in the reaction mixture, in agreement with the high copolymer yields. The ^1^H NMR spectra of the various copolymers correspond to the spectrum of **PNVCL** homopolymer, as exemplified for **PNVCL-g** in Figure 2. The apparent molar masses of the various copolymers were all similar, being within the range of 70,000 to 120,000.

### 3.2. Spectroscopic Properties of the Monomers and Polymers In Ethanol

**Figure 3:**
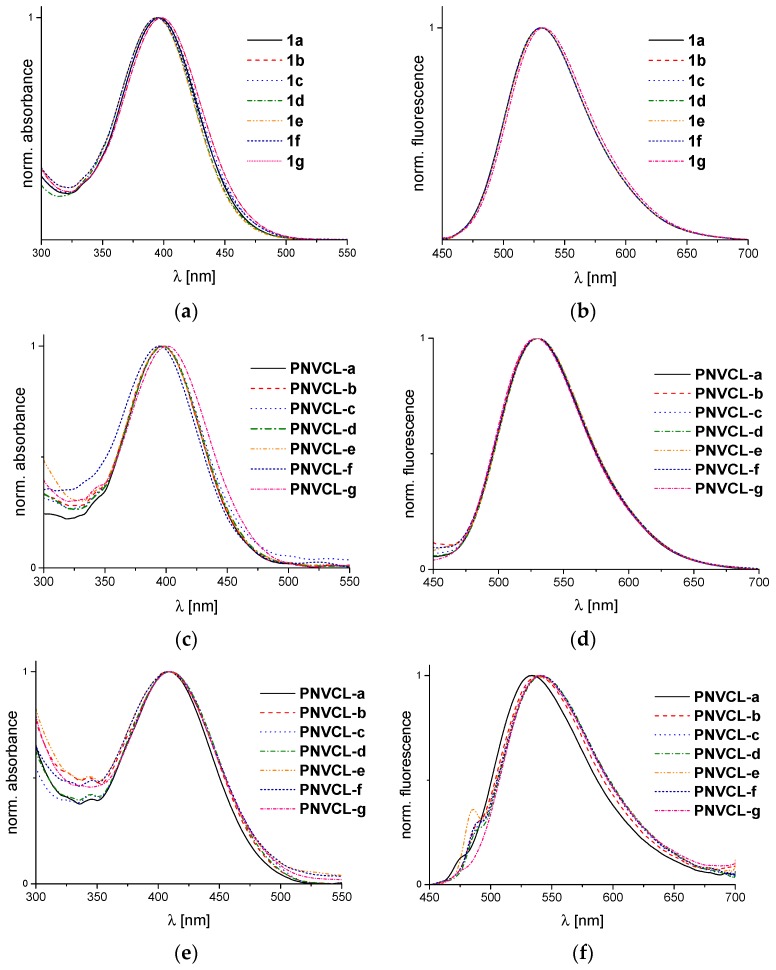
Normalized UV-VIS and fluorescence spectra of dye monomers and dye-labeled copolymers in solution (21 °C): (**a**) absorbance spectra of dye monomers **1a**–**1g** in ethanol; (**b**) emission spectra of dye monomers **1a**–**1g** in ethanol (λ^Exc^ = 400 nm); (**c**) absorbance spectra of copolymers **PNVCL-a**–**PNVCL-g** in ethanol; (**d**) emission spectra of copolymers **PNVCL-a**–**PNVCL-g** in ethanol (λ^Exc^ = 396 nm); (**e**) absorbance spectra of copolymers **PNVCL-a**–**PNVCL-g** in water; and (**f**) emission spectra of copolymers **PNVCL-a**–**PNVCL-g** in water (λ^Exc^ = 409–418 nm).

As dye monomers, **1a**–**1g** were virtually insoluble in water, spectroscopic properties of the markedly solvatochromic monomers and polymers were first characterized in ethanol, as illustrated in Figure 3. Table 2 summarizes the key values for the monomers derived therefrom. Typically, they show absorbance maxima in the range of 395–398 nm, and emission maxima in the range of 530–534 nm (Figure 3a,b). Accordingly, the fluorescence spectra exhibit pronounced Stoke shifts. The quantum yields are low in the polar protic solvent ethanol. The latter findings correspond well to a recent report on the fluorescence of a methacrylate dye monomer bearing the closely related fluorophore 4-(*N*,*N*-dimethylamino)-1,8-naphthalimide [21].

Still, despite the chemically very similar structure of the 4-(morpholino)-1,8-naphthalimide fluorophore used here, all monomers **1a**–**1g** exhibit a blue shift of about 20–25 nm for the absorbance band in comparison to the previously studied system, suggesting a notably weaker electron donor effect of the morpholino compared to the dimethylamino substituent, while the emission band is hypsochromically shifted by only 5 nm.

The homopolymer **PNVCL‑0** as well as all copolymers of **NVCL** with the various dye monomers, *i.e*, **PNVCL-a**–**PNVCL-g**, were soluble in both ethanol and water at ambient temperature. For comparison with the monomers, their spectroscopic properties were first characterized in ethanol (Figure 3c,d). Similar to the monomers, all absorbance maxima λ_max_^Abs^ are within the range of 395–398 nm, while emission maxima λ_max_^Em^ are all 530 ± 1 nm, as summarized in Table 3. Compared to the behavior of the monomers (see Table 2), the absorbance maxima of the chromophores seem hardly changed by incorporation into PNVCL, while for the emission maxima, a small but systematic hypsochromic shift of *ca.* 2 nm seems to occur.

The spectroscopic properties of the polymers were additionally investigated in water (Figure 3e,f), in which the monomers are insoluble. All polymers display very similar absorbance spectra, in which the maxima are shifted bathochromically by about 10 nm in comparison to ethanol solutions. In addition, concerning the fluorescence spectra, we note a general bathochromic shift of the emission maxima in water compared to ethanol, which is in the order of 10 nm. The general red shift encountered of both the absorbance and emission spectra in water compared to the less polar solvent ethanol, is in agreement with the established solvatochromic behavior of the 4-amino-1,8-naphthalimide chromophore [21,68]. The emission maxima of the various dye-labeled polymers in water are all located closely around 540 nm, with the exception of **PNVCL-a** for which λ_max_^Em^ is blue-shifted to about 534 nm. This copolymer bears the most hydrophobic substituent, namely an octadecyl chain, in the direct neighborhood of the fluorophore, thus apparently reducing the local polarity somewhat.

Noteworthy, a close analysis of the shape of the emission bands of the polymers in aqueous solution reveals small shoulders, and in the case of **PNVCL-e** even a small secondary maximum on the low wavelength slope of the bands. This points to the formation of H-aggregates, presumably due to the hydrophobic character of the fluorophore, despite of the low dye content. As these shoulders are very small, the role of the presumably underlying aggregates was supposed to be negligible for the studies of the thermo-responsive behavior (see below). Still, the finding underlines that the potential risk of hydrophobic aggregation of environmentally sensitive dyes has always to be kept in mind when designing and analyzing polymeric dye based molecular thermometers.

### 3.3. Thermoresponsive Behavior of the Dye-Labeled Polymers

**Figure 4:**
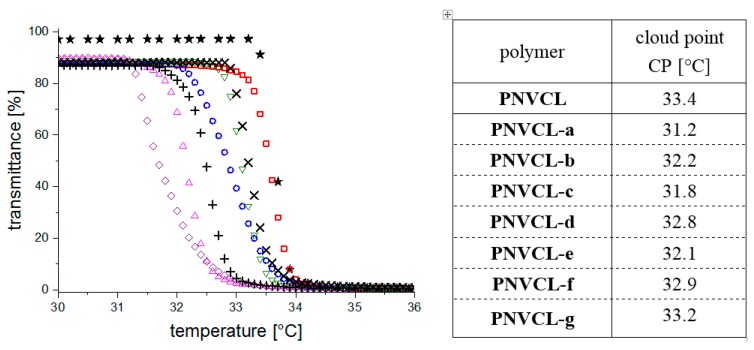
Temperature dependent turbidimetric analysis of **PNVCL** and dye-labeled **NVCL** copolymers in aqueous solutions (3.00 g·L^−1^, heating rate 0.2 K·min^−^). (★) = **PNVCL**, (◇) = **PNVCL-a,** (o) = **PNVCL-b,** (Δ) = **PNVCL-c,** (▽) = **PNVCL-d,** (＋) = **PNVCL-e,** (×) = **PNVCL-f,** (□) = **PNVCL-g**. The phase transition temperatures were taken as the temperature of the onset of clouding (cloud points CP).

The cloud point, *i.e.*, the phase transition temperature, of the reference homopolymer **PNVCL-0** in aqueous solution was found by turbidimetry as 33.7 °C at a concentration of 3 g·L^−1^ (Figure 4). The hysteresis observed for heating and cooling curves were about 1 °C or less, and thus, virtually negligible. The cloud point value agrees well with literature data on comparably concentrated aqueous solutions of **PNVCL** samples having similar molar mass [54,56,58,59].

Similarly for polymers **PNVCL-a**–**PNVCL-g**, the cloud points were all in the range of 31–33.5 °C (Figure 4). As expected, the incorporation of small amounts of a functional comonomer did not interfere much with the thermo-responsiveness of the parent homopolymer PNVCL. Still, we find that all copolymers, which contain in average less than 1 comonomer unit per polymer chain, exhibit a small, but nevertheless notable decrease of the cloud point of 0.5 to 2.5 °C compared to **PNVCL-0**, the homopolymer reference. One might be tempted to correlate the reduced cloud points directly to the hydrophobicity of the dye monomer units incorporated, in particular to the relative differences in the distinct hydrophobic character of the substituents attached to the second carboxyl group of the maleic acid moiety. Indeed, **PNVCL-a** incorporating the most hydrophobic comonomer **1a**, shows the lowest cloud point within the series. However, the situation is more complex, as the most hydrophilic substituents, as found in monomers **1d** and **1f**, do not provoke the highest cloud points of the copolymer series. In fact, it seems that the small differences seen additionally reflect the differences in molar mass of the samples (see Table 1). Accordingly, the slightly lower cloud points of the dye-labeled copolymers result from combined effects of the somewhat increased molar masses and the somewhat increased overall hydrophobicity. Anyhow, the phase transitions of the various polymers prepared occur within a very narrow temperature window, and thus enable direct comparative studies of the temperature induced spectroscopic changes.

**Figure 5:**
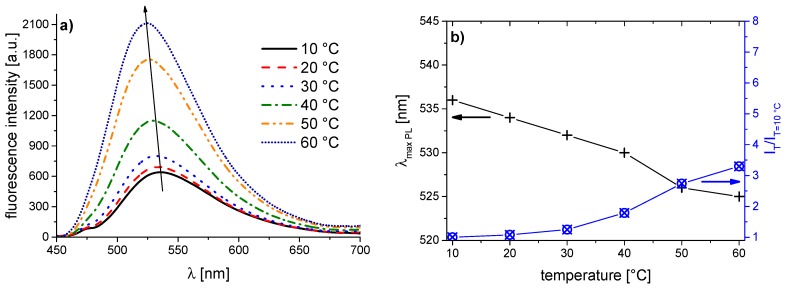
Temperature dependent fluorescence behavior of **PNVCL-a** in aqueous solution (concentration 0.10 g·L^−1^): (**a**) normalized emission spectra (λ^Exc^ = 418 nm); and (**b**) evolution of λ_max_^Em^ of the emission band, and of the fluorescence intensity normalized to 10 °C.

**Figure 6:**
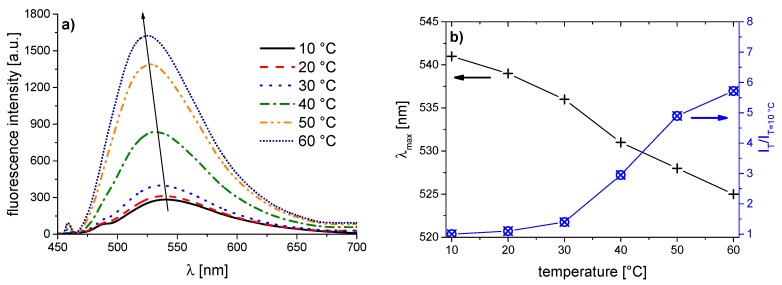
Temperature dependent fluorescence behavior of **PNVCL-b** in aqueous solution (concentration 0.10 g·L^−1^): (**a**) normalized emission spectra (λ^Exc^ = 418 nm); and (**b**) evolution of λ_max_^Em^ of the emission band, and of the fluorescence intensity normalized to 10 °C.

**Figure 7:**
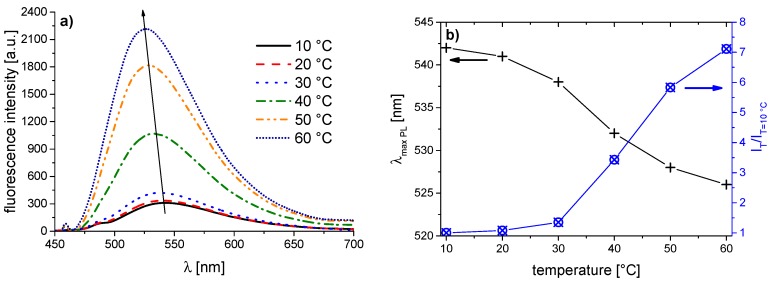
Temperature dependent fluorescence behavior of **PNVCL-c** in aqueous solution (concentration 0.10 g·L^−1^): (**a**) normalized emission spectra (λ^Exc^ = 418 nm); and (**b**) evolution of λ_max_^Em^ of the emission band, and of the fluorescence intensity normalized to 10 °C.

**Figure 8:**
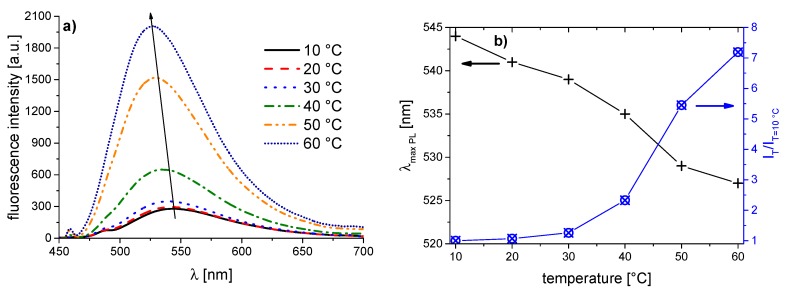
Temperature dependent fluorescence behavior of **PNVCL-d** in aqueous solution (concentration 0.10 g·L^−1^): (**a**) normalized emission spectra (λ^Exc^ = 418 nm); and (**b**) evolution of λ_max_^Em^ of the emission band, and of the fluorescence intensity normalized to 10 °C.

**Figure 9:**
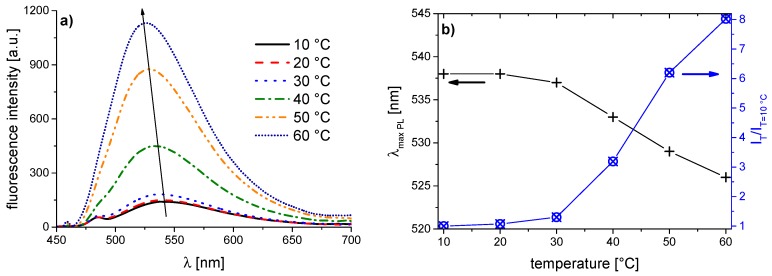
Temperature dependent fluorescence behavior of **PNVCL-e** in aqueous solution (concentration 0.10 g·L^−1^):(**a**) normalized emission spectra (λ^Exc^ = 416 nm); and (**b**) evolution of λ_max_^Em^ of the emission band, and of the fluorescence intensity normalized to 10 °C.

**Figure 10:**
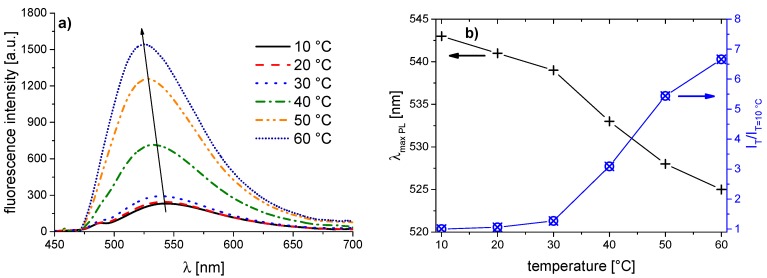
Temperature dependent fluorescence behavior of **PNVCL-f** in aqueous solution (concentration 0.10 g·L^−1^): (**a**) normalized emission spectra (λ^Exc^ = 418 nm); and (**b**) evolution of λ_max_^Em^ of the emission band, and of the fluorescence intensity normalized to 10 °C.

**Figure 11:**
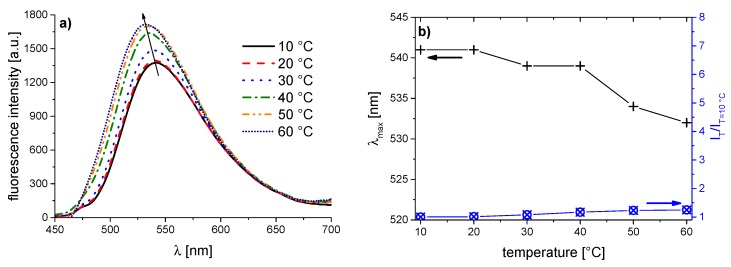
Temperature dependent fluorescence behavior of **PNVCL-g** in aqueous solution (concentration 0.10 g·L^−1^): (**a**) normalized emission spectra (λ^Exc^ = 409 nm); and (**b**) evolution of λ_max_^Em^ of the emission band, and of the fluorescence intensity normalized to 10 °C.

The corresponding evolution of the fluorescence spectra of the copolymer series with increasing temperature is illustrated in Figure 5, Figure 6, Figure 7, Figure 8, Figure 9, Figure 10 and Figure 11. We find for all dye-labeled polymers marked changes of their fluorescence behavior with the temperature. Qualitatively, we observe a marked increase of the fluorescence intensity when passing through the phase transition. In addition, we note a marked hypsochromic shift of the emission maximum, indicating a less polar environment of the chromophores above the phase transition. Accordingly, all dye-labeled polymers may act *a priori* as molecular thermometers.

Nevertheless, the quantitative fluorescence responses to temperature changes differ substantially between the individual polymers. Clearly, the thermally-induced spectral shift as well as the induced fluorescence enhancement are by far the smallest for **PNVCL-g** (Figure 11). More pronounced, but still relatively weak effects are observed in the case of **PNVCL-a** (Figure 5). The strongest fluorescence enhancement is seen for samples **PNVCL-d** and **PNVCL-e**, while the highest blue shifts are encountered for samples **PNVCL-d** and **PNVCL-f** (Figure 8, Figure 9 and Figure 10).

The key data extracted from the various plots are summarized in Table 4. When correlating the data with the chemical structure of the dye comonomers incorporated, we see that the by far weakest effects of the phase transition are seen when incorporating comonomer **1g**. In this comonomer, the chromophore is attached to the polymer backbone by a short spacer only. As the caprolactam side chains are amphiphilic, and in particular, present a rather large hydrophobic fragment in the vicinity of the naphthalimide fluorophore, one may attribute this finding to an inherent partial hydrophobic shielding of the fluorophore. Such a shielding is effective also at temperatures well below the phase transition. A similar explanation was proposed for explaining the weak fluorescence enhancement of incorporated naphthalimide dyes in poly(oligoethyleneglycol acrylate)s above the phase transition of the LCST-type. Alternatively, the short spacer group possibly precludes the efficient transfer of the fluorophore from an aqueous environment to the hydrophobic pockets formed by the collapsed polymer coils above the phase transition, thus exposing the label to a partially hydrated environment even at high temperatures. The latter explanation is supported by the spectral data (see Table 3). While the value of λ_max_^Em^ of 541 nm for **PNVCL-g** below the cloud point is indicative of a well-hydrated environment, λ_max_^Em^ reaches only a value of 532 nm above the phase transition temperature, which indicates a still rather polar environment.

The situation becomes much more favorable, when a longer spacer is incorporated between label and polymer backbone (as in copolymers **PNVCL-a**–**PNVCL-f**), which enables the fluorophore “to stick out” beyond the side chains of the polymer matrix, and simultaneously renders it more mobile. However, the still moderate effects found for **PNVCL-a** exemplify, that overly big hydrophobic fragments attached to the polymer reduce the sensitivity of the label to the conformational changes nevertheless, and thus, obscure notably the subsequent changes of the local environment. This explanation would fit with the relatively low value for λ_max_^Em^ of 536 nm at 10 °C, pointing to a partially dehydrated environment already at low temperatures.

In reverse, hydrophilic moieties attached in the vicinity of the dye label improve the fluorescence response, as seen by the very strong spectral as well as intensity changes encountered for polymers **PNVCL-d**, **PNVCL-e**, and **PNVCL-f**. They bear the most hydrophilic side groups fixed to the maleamide comonomers. Note, that for the copolymers with the long spacer separating the label from the backbone, even moderately hydrophobic substituents, as introduced by comonomers **1b** and **1c**, still allow for strong thermally-induced fluorescence effects.

Interestingly, the closer analysis of the data in Table 4 also reveals, that the extents of fluorescence enhancement and of induced spectral shifts do not strictly coincide. Copolymer **PNVCL-e**, which shows the highest increase of fluorescence when crossing the phase transition, provides a spectral shift of 12 nm only, which is clearly below the shifts of 16–18 nm achieved by **PNVCL-b**–**PNVCL-d** and **PNVCL-f**. At present, we can only speculate about this at the first sight somewhat puzzling observation. Possibly, the particularly long spacer group in comonomer **1e**, due to the build up by the azide-alkyne “click reaction”, places the fluorophore and the hydrophilic fragment very far apart. This enables the fluorophore to avoid the neighborhood of the hydrophilic group effectively, and to stay close to the more hydrophobic lactam side chains of the polymer matrix. This explanation would be consistent with the relatively low value observed for λ_max_^Em^ of 538 nm at 10 °C, pointing to a partially dehydrated environment already at low temperatures, as discussed above for the case of **PNVCL-a**. In contrast, the local polarity sensed above the phase transition is comparable to the ones found for the other copolymers (see Table 4).

Concerning temperature-regulated fluorescence effects, these findings suggest that one has to distinguish between effects due to a coil-to-globule collapse induced change of the average dielectric constant of the fluorophore’s direct vicinity (governing the positions of the absorbance and emission bands, and thus, the extent of the solvatochromic shifts), and of the number of solvent molecules in direct contact to the fluorophore (that act as quenchers and govern the quantum yield). Accordingly, an appropriate design for efficient polymeric molecular thermometers asks for the separation of the fluorophore from the backbone by a spacer group of a specific length, in addition to the appropriate selection of the environmentally sensitive fluorophore. Moreover, it seems that hydrophilic groups fixed in the vicinity of the fluorophores are advantageous, as they increase the difference felt between the well-hydrated state below the phase transition temperature, and the—more or less—dehydrated state above, thus increasing the thermometer’s sensitivity.

Another instructive feature evident from Figure 5, Figure 6, Figure 7, Figure 8, Figure 9, Figure 10 and Figure 11 are the differing temperature profiles of the fluorescence intensity changes and of the induced solvatochromic shifts. The latter extend systematically over a considerably broader temperature range. This implies that for monitoring precisely very small temperature changes, fluorescence intensity will be the more sensitive parameter to follow, whereas for monitoring a larger temperature window, the solvatochromic shift will be the more suited parameter. In any case, the simultaneous monitoring of both parameters enables to crosscheck the temperature value deduced, thus enhancing the reliability of the method.

## 4. Conclusions

Naphthalimide functionalized maleic acid diamides or esteramides bearing various hydrophilic or hydrophobic substituents are useful functional comonomers for the copolymerization with vinylamides such as *N*-vinylcaprolactam. The resulting dye-labeled copolymers are thermo-sensitive in aqueous solution, and behave as molecular thermometers. On the one hand, the fluorescence intensity of the polymers undergoes a dramatic increase when passing from below to above the phase transition temperature. On the other hand, the emission band maximum of the solvatochromic fluorophores incurs a marked hypsochromic shift. Both parameters enable independently to follow the temperature of the system in a window of about 10 or 40 K, respectively, around the phase transition temperature. Interestingly, the temperature regulated responses of the two effects, *i.e.*, the extent of the modulation of the fluorescence intensity and of the solvatochromic shift encountered, do not coincide exactly. In addition, they cover temperature windows of differing widths. Still, they may be exploited jointly, to improve the reliability of such spectroscopic temperature measurements.

The broad variation of the substituents undertaken on the maleic comonomers provides some guidelines for the molecular design of effective polymeric molecular thermometers. First of all, the studies demonstrate the importance of a spacer group of a specific length that separates the dye label from the polymer backbone. An appropriate spacer seems essential to obtain sensitive temperature regulated fluorescence responses. Accordingly, the successful realization of effective polymeric molecular thermometers based on PNIPAM in the past seems serendipitous, as the side chains of PNIPAM are small, and therefore, the spacer group can be very short. In addition to the need for a spacer group, it seems that hydrophilic groups fixed in the vicinity of the fluorophore label help to increase the difference felt between the well hydrated state below, and, respectively, the less hydrated state above the phase transition temperature, thus increasing the thermometer’s sensitivity.

## Figures and Tables

**Table 1:** Copolymers of *N*-vinylcaprolactam (**NVCL**) made with 0.1 mol % of functional maleamides and 1 mol % of initiator in the reaction mixture, and their analytical data.

Polymer	Comonomer	Yield (%)	Dye monomer incorporated (mol %) ^a^	*M*_n_^app^ (kDa) ^b^	*M*_w_^app^ (kDa) ^b^	Average No. of fluorophores per polymer chain
**PNVCL-0**	-	82 ^c^	-	30	72	-
**PNVCL-a**	1a	72 ^d^	0.10	30	119	0.2
**PNVCL-b**	1b	82 ^d^	0.09	24	100	0.2
**PNVCL-c**	1c	76 ^d^	0.12	47	125	0.4
**PNVCL-d**	1d	70 ^d^	0.10	21	75	0.2
**PNVCL-e**	1e	70 ^d^	0.11	48	117	0.4
**PNVCL-f**	1f	80 ^c^	0.07	26	93	0.1
**PNVCL-g**	1g	80 ^c^	0.16	25	86	0.3

^a^ calculated from the absorbance maximum in the VIS spectra, assuming identical molar absorption coefficients for the chromophore in the monomer and copolymer; ^b^ by size exclusion chromatography in DMF, calibrated with polystyrene standards; ^c^ initiator AIBN, ^d^ initiator V-501.

**Table 2:** Photophysical characteristics of dye monomers **1a**‑**1g** in ethanol (21 °C).

Dye monomer	λ_max_^Abs^ (EtOH) (nm)	ε (EtOH) (L·mol^−1^·cm^−1^)	λ_max_^Em^ (EtOH) (nm)	Φ (EtOH) (%)
**1a**	396	10,000	532	0.09
**1b**	398	11,000	532	0.07
**1c**	396	8,800	530	0.07
**1d**	396	9,700	531	0.08
**1e**	395	9,700	532	0.09
**1f**	395	9,400	531	0.08
**1g**	398	9,400	534	0.06

**Table 3:** Photophysical characteristics of dye-labeled polymers **PNVCL-a**–**PNVCL-g** in ethanol and water (21 °C).

Polymer	λ_max_^Abs^ (EtOH) (nm)	λ_max_^Em^ (EtOH) (nm)	λ_max_^Abs^ (H_2_O) (nm)	λ_max_^Em^ (H_2_O) (nm)
**PNVCL-a**	397	530	407	534
**PNVCL-b**	397	530	410	539
**PNVCL-c**	398	530	410	541
**PNVCL-d**	397	530	410	541
**PNVCL-e**	397	530	407	538
**PNVCL-f**	395	530	409	541
**PNVCL-g**	400	529	410	541

**Table 4:** Spectral characteristics of the dye-labeled PNVCL polymers in aqueous solution (concentration 0.10 g·L^−1^).

Polymer	λ_max_^Abs^ (nm)	λ_max_^Em^ at 10 °C (nm)	λ_max_^Em^ at 60 °C (nm)	Δλ_max_^10–60 °C^ (nm) ^a^	I_T=60°C_/I_T=10°C_^b^
**PNVCL-a**	407	536	525	11	3.3
**PNVCL-b**	410	541	525	16	5.7
**PNVCL-c**	410	542	526	16	6.6
**PNVCL-d**	410	543	527	16	7.2
**PNVCL-e**	407	538	526	12	8.0
**PNVCL-f**	408	543	525	18	6.7
**PNVCL-g**	410	541	532	10	1.2

^a^ solvatochromic shift of the emission maximum between 10 and 60 °C; ^b^ difference of the fluorescence intensities at 10 and 60 °C.

## References

[1] Liu R., Fraylich M., Saunders B.R. (2009). Thermoresponsive copolymers: From fundamental studies to applications. Colloid Polym. Sci..

[2] Wischerhoff E., Badi N., Lutz J.-F., Laschewsky A. (2010). Smart Bioactive Surfaces. Soft Matter.

[3] Aseyev V., Tenhu H., Winnik F. (2011). Non-ionic thermoresponsive polymers in water. Adv. Polym. Sci..

[4] Ward M.A., Georgiou T.K. (2011). Thermoresponsive polymers for biomedical applications. Polymers.

[5] Hoogenboom R., Schlaad H. (2011). Bioinspired poly(2-oxazoline)s. Polymers.

[6] Roy D., Brooks W.L.A., Sumerlin B.S. (2013). New directions in thermoresponsive polymers. Chem. Soc. Rev..

[7] Aseyev V., Tenhu H., Winnik F. (2006). Temperature dependence of the colloidal stability of neutral amphiphilic polymers in water. Adv. Polym. Sci..

[8] Ringsdorf H., Venzmer J., Winnik F. (1991). Interaction of hydrophobically-modified poly-*N*-isopropylacrylamides with model membranes—Or playing a molecular accordion. Angew. Chem. Int. Ed..

[9] Mertoglu M., Garnier S., Laschewsky A., Skrabania K., Storsberg J. (2005). Stimuli responsive amphiphilic block copolymers for aqueous media synthesised via reversible addition fragmentation chain transfer polymerisation (RAFT). Polymer.

[10] Birnbaum W., Kuckling D. (2012). Synthesis of α-biotinyl poly(ethylene glycol-*b*-*N*-isopropylacrylamide) block copolymers with different fluorescent dyes at the ω-side. Polym. Chem..

[11] Hiruta Y., Shimamura M., Matsuura M., Maekawa Y., Funatsu T., Suzuki Y., Ayano E., Okano T., Kanazawa H. (2014). Temperature-responsive fluorescence polymer probes with accurate thermally controlled cellular uptakes. ACS Macro Lett..

[12] Winnik F.M., Ottaviani M.F., Bossman S.H., Pan W., Garcia-Garibay M., Turro N.J. (1993). Phase separation of poly(*N*-isopropylacrylamide) in water: A spectroscopic study of a polymer tagged with a fluorescent dye and a spin label. J. Phys. Chem..

[13] Laukkanen A., Winnik F.M., Tenhu H. (2005). Pyrene-labeled graft copolymers of *N*-vinylcaprolactam: Synthesis and solution properties in water. Macromolecules.

[14] Yoshinari E., Furukawa H., Horie K. (2005). Fluorescence study on the mechanism of rapid shrinking of grafted poly(*N*-isopropylacrylamide) gels and semi-IPN gels. Polymer.

[15] Chee C.K., Rimmer S., Soutar I., Swanson L. (2006). Fluorescence investigations of the conformational behaviour of poly(*N*-vinylcaprolactam). React. Funct. Polym..

[16] Matsumura Y., Katoh A. (2008). Synthesis of 2,3-dimorpholino-6-aminoquinoxaline derivatives and application to a new intramolecular fluorescent probe. J. Lumin..

[17] Yusa S.-I., Endo T., Ito M. (2009). Synthesis of thermo-responsive 4-arm star-shaped porphyrin-centered poly(*N*,*N*-diethylacrylamide) via reversible addition-fragmentation chain transfer radical polymerization. J. Polym. Sci. A Polym. Chem..

[18] Nagai A., Kokado K., Miyake J., Cyujo Y. (2010). Thermoresponsive fluorescent water-soluble copolymers containing BODIPY dye: Inhibition of H-aggregation of the BODIPY units in their copolymers by LCST. J. Polym. Sci. A Polym. Chem..

[19] Weiss J., Laschewsky A. (2011). Temperature induced self-assembly of triple responsive triblock copolymers in aqueous solutions. Langmuir.

[20] Thivaios I., Diamantis I., Bokias G., Kallitsis J.K. (2012). Temperature-responsive photoluminescence of quinoline-labeled poly(*N*-isopropylacrylamide) in aqueous solution. Eur. Polym. J..

[21] Inal S., Kölsch J.D., Chiappisi L., Janietz D., Gradzielski M., Laschewsky A., Neher D. (2013). Structure-related differences in the temperature regulated fluorescence response of LCST type polymers. J. Mater. Chem. C.

[22] Jordão N., Gavara R., Parola A.J. (2013). Flavylium-supported poly(*N*-isopropylacrylamide): A class of multistimuli responsive polymer. Macromolecules.

[23] Wu Y., Hu H., Hu J., Liu T., Zhang G., Liu S. (2013). Thermo- and light-regulated formation and disintegration of double hydrophilic block copolymer assemblies with tunable fluorescence emissions. Langmuir.

[24] Park Y.I., Zhang B., Kuo C.-Y., Martinez J.S., Park J., Mallapragada S., Wang H.-L. (2013). Stimuli-responsive poly-*N*-isopropylacrylamide: Phenylene vinylene Oligomer Conjugate. J. Phys. Chem. C.

[25] Uchiyama S., Matsumura Y., de Silva A.P., Iwai K. (2003). Fluorescent molecular thermometers based on polymers showing temperature-induced phase transitions and labeled with polarity-responsive benzofurazans. Anal. Chem..

[26] Uchiyama S., Matsumura Y., Prasanna de Silva A., Iwai K. (2004). Modulation of the sensitive temperature range of fluorescent molecular thermometers based on thermoresponsive polymers. Anal. Chem..

[27] Iwai K., Matsumura Y., Uchiyama S., Prasanna de Silva A. (2005). Development of fluorescent microgel thermometers based on thermo-responsive polymers and their modulation of sensitivity range. J. Mater. Chem..

[28] Matsumura Y., Iwai K. (2005). Synthesis and thermo-responsive behavior of fluorescent labeled microgel particles based on poly(*N*-isopropylacrylamide) and its related polymers. Polymer.

[29] Shiraishi Y., Miyamoto R., Zhang X., Hirai T. (2007). Rhodamine-based fluorescent thermometer exhibiting selective emission enhancement at a specific temperature range. Org. Lett..

[30] Shiraishi Y., Miyamoto R., Hirai T. (2008). A Hemicyanine-conjugated copolymer as a highly sensitive fluorescent thermometer. Langmuir.

[31] Gota C., Uchiyama S., Yoshihara T., Tobita S., Ohwada T. (2008). Temperature-dependent fluorescence lifetime of a fluorescent polymeric thermometer, poly(*N*-isopropylacrylamide), Labeled by polarity and hydrogen bonding sensitive 4-sulfamoyl-7-aminobenzofurazan. J. Phys. Chem. B.

[32] Gota C., Okabe K., Funatsu T., Harada Y., Uchiyama S. (2009). Hydrophilic fluorescent nanogel thermometer for intracellular thermometry. J. Am. Chem. Soc..

[33] Guo Z., Zhu W., Xiong Y., Tian H. (2009). Multiple logic fluorescent thermometer system based on *N*-isopropylmethacrylamide copolymer bearing dicyanomethylene-4*H*-pyran moiety. Macromolecules.

[34] Pietsch C., Hoogenboom R., Schubert U.S. (2009). Soluble polymeric dual sensor for temperature and pH value. Angew. Chem. Int. Ed..

[35] Wang D., Miyamoto R., Shiraishi Y., Hirai T. (2009). BODIPY-conjugated thermoresponsive copolymer as a fluorescent thermometer based on polymer microviscosity. Langmuir.

[36] Nagai A., Yoshii R., Otsuka T., Kokado K., Chujo Y. (2010). BODIPY-based chain transfer agent: Reversibly thermoswitchable luminescent gold nanoparticle stabilized by BODIPY-terminated water-soluble polymer. Langmuir.

[37] Chen C.-Y., Chen C.-T. (2011). A PNIPAM-based fluorescent nanothermometer with ratiometric readout. Chem. Commun..

[38] Hu J., Zhang X., Wang D., Hu X., Liu T., Zhang G., Liu S. (2011). Ultrasensitive ratiometric fluorescent pH and temperature probes constructed from dye-labeled thermoresponsive double hydrophilic block copolymers. J. Mater. Chem..

[39] Uchiyama S., Kimura K., Gota C., Okabe K., Kawamoto K., Inada N., Yoshihara T., Tobita S. (2012). Environment-sensitive fluorophores with benzothiadiazole and benzoselenadiazole structures as candidate components of a fluorescent polymeric thermometer. Chem. Eur. J..

[40] Guo Y., Yu X., Xue W., Huang S., Dong J., Wei L., Maroncelli M., Li H. (2014). Synthesis, structures, and properties of a fluoranthene-based biphenol polymer as a fluorescent nano-thermometer. Chem. Eng. J..

[41] Qiao J., Chen C., Qi L., Liu M., Dong P., Jiang Q., Yang X., Mu X., Mao L. (2014). Intracellular temperature sensing by a ratiometric fluorescent polymer thermometer. J. Mater. Chem. B.

[42] Zhou J., Mishra K., Bhagat V., Joy A., Becker M.L. (2015). Thermoresponsive dual emission nanosensor based on quantum dots and dye labeled poly(*N*-isopropylacrylamide). Polym. Chem..

[43] Malfait A., Coumes F., Fournier D., Cooke G., Woisel P. (2015). A water-soluble supramolecular polymeric dual sensor for temperature and pH with an associated direct visible readout. Eur. Polym. J..

[44] Vancoillie G., Zhang Q., Hoogenboom R. (2016). Chapter 7: Polymeric temperature sensors. Thermometry at the Nanoscale: Techniques and Selected Applications.

[45] Koopmans C., Ritter H. (2007). Color change of *N*-isopropylacrylamide copolymer bearing reichardts dye as optical sensor for lower critical solution temperature and for host-guest interaction with β-cyclodextrin. J. Am. Chem. Soc..

[46] Hu J., Dai L., Liu S. (2011). Analyte-reactive amphiphilic thermoresponsive diblock copolymer micelles-based multifunctional ratiometric fluorescent chemosensors. Macromolecules.

[47] Pietsch C., Schubert U.S., Hoogenboom R. (2011). Aqueous polymeric sensors based on temperature-induced polymer phase transitions and solvatochromic dyes. Chem. Commun..

[48] Zhou X., Su F., Tian Y., Johnson R.H., Meldrum D.R. (2011). Platinum (II) porphyrin-containing thermoresponsive poly(*N*-isopropylacrylamide) copolymer as fluorescence dual oxygen and temperature sensor. Sens. Actuators. B Chem..

[49] Inal S., Chiappisi L., Kölsch J.D., Kraft M., Appavou M.-S., Scherf U., Wagner M., Hansen M.R., Gradzielski M., Laschewsky A. (2013). Temperature-regulated fluorescence and association of an oligo(ethyleneglycol)-methacrylate-based copolymer with a conjugated polyelectrolyte—The effect of solution ionic strength. J. Phys. Chem. B.

[50] Inal S., Kölsch J.D., Sellrie F., Schenk J.A., Wischerhoff E., Laschewsky A., Neher D. (2013). A water soluble fluorescent polymer as a dual colour sensor for temperature and a specific protein. J. Mater. Chem. B.

[51] Seeboth A., Lötzsch D., Ruhmann R., Muehling O. (2014). Thermochromic polymers—Function by design. Chem. Rev..

[52] Eisele M., Burchard W. (1990). Hydrophobic water-soluble polymers, 1. Dilute solution properties of poly(1-vinyl-2-piperidone) and poly(*N*-vinylcaprolactam). Makromol. Chem..

[53] Tager A.A., Safronov A.P., Sharina S.V., Galaev I.Y. (1993). Thermodynamic study of poly(*N*-vinyl caprolactam) hydration at temperatures close to lower critical solution temperature. Colloid Polym. Sci..

[54] Meeussen F., Nies E., Berghmans H., Verbrugghe S., Goethals E., Du Prez F. (2000). Phase behaviour of poly(*N*-vinyl caprolactam) in water. Polymer.

[55] Aseyev V., Hietala S., Laukkanen A., Nuopponen M., Confortini O., Du Prez F.E., Tenhu H. (2005). Mesoglobules of thermoresponsive polymers in dilute aqueous solutions above the LCST. Polymer.

[56] Zhao X., Coutelier O., Nguyen H.H., Delmas C., Destarac M., Marty J.-D. (2015). Effect of copolymer composition of RAFT/MADIX-derived *N*-vinylcaprolactam/*N*-vinylpyrrolidone statistical copolymers on their thermoresponsive behavior and hydrogel properties. Polym. Chem..

[57] Lozinsky V.I., Simenel I.A., Kurskaya E.A., Kulakova V.K., Galaev I.Y., Mattiasson B., Grinberg V.Y., Grinberg N.V., Khokhlov A.R. (2000). Synthesis of *N*-vinylcaprolactam polymers in water-containing media. Polymer.

[58] Ieong N.S., Hasan M., Phillips D.J., Saaka Y., O’Reilly R.K., Gibson M.I. (2012). Polymers with molecular weight dependent LCSTs are essential for cooperative behaviour. Polym. Chem..

[59] Shao L., Hu M., Chen L., Xu L., Bi Y. (2012). RAFT polymerization of *N*-vinylcaprolactam and effects of the end group on the thermal response of poly(*N*-vinylcaprolactam). React. Funct. Polym..

[60] Vihola H., Laukkanen A., Valtola L., Tenhu H., Hirvonen J. (2005). Cytotoxicity of thermosensitive polymers poly(*N*-isopropylacrylamide), poly(*N*-vinylcaprolactam) and amphiphilically modified poly(*N*-vinylcaprolactam). Biomaterials.

[61] Anton P., Laschewsky A. (1993). Zwitterionic polysoaps with reduced density of surfactant side groups. Makromol. Chem..

[62] El-Guweri M., Hendlinger P., Laschewsky A. (1997). Partially fluorinated maleimide copolymers for Langmuir films of improved stability, 1. Synthesis, copolymerisation behaviour and bulk properties. Macromol. Chem. Phys..

[63] Auzély-Velty R., Cristea M., Rinaudo M. (2002). Galactosylated *N*-vinylpyrrolidone-maleic acid copolymers: Synthesis, characterization, and interaction with lectins. Biomacromolecules.

[64] Popescu I., Prisacaru A.I., Suflet D.M., Fundueanu G. (2014). Thermo- and pH-sensitivity of poly(*N*-vinylcaprolactam-*co*-maleic acid) in aqueous solution. Polym. Bull..

[65] Dahmén J., Frejd T., Grönberg G., Lave T., Magnusson G., Noori G. (1983). 2-Bromoethyl glycosides: Synthesis and characterisation. Carbohydr. Res..

[66] Kleinert M., Röckendorf N., Lindhorst T.K. (2004). Glyco-SAMs as glycocalyx mimetics: Synthesis of L-Fucose- and D-Mannose-terminated building blocks. Eur. J. Org. Chem..

[67] Wu J., Yi T., Shu T., Yu M., Zhou Z., Xu M., Zhou Y., Zhang H., Han J., Li F. (2008). Ultrasound switch and thermal self-repair of morphology and surface wettability in a cholesterol-based self-assembly system. Angew. Chem., Int. Ed..

[68] Yang Z., Cao J., He Y., Yang J.H., Kim T., Peng X., Kim J.S. (2014). Macro-/micro-environment-sensitive chemosensing and biological imaging. Chem. Soc. Rev..

